# Integrating Desert Sand Utilization in Saltwater Aqua-Vegeculture Production: Performance Evaluation of Yield and Biochemical Composition

**DOI:** 10.3390/ani15091246

**Published:** 2025-04-28

**Authors:** Radhakrishnan Subramanian, Chythra Somanathan Nair, Ramya Manoharan, Drishya Nishanth, Abdul Jaleel

**Affiliations:** 1 ASPIRE Research Institute for Food Security in the Drylands (ARIFSID), United Arab Emirates University, Al Ain P.O. Box 15551, United Arab Emirates; drsrk@uaeu.ac.ae (R.S.); 201970194@uaeu.ac.ae (D.N.); 2 Department of Integrative Agriculture, College of Agriculture and Veterinary Medicine, United Arab Emirates University, Al Ain P.O. Box 15551, United Arab Emirates; chythra_n@uaeu.ac.ae (C.S.N.); 700039316@uaeu.ac.ae (R.M.)

**Keywords:** saltwater aquaponics, tilapia fish, lettuce, iAVs, biochemical composition

## Abstract

Background: Saline aquaponics integrates fish and crop cultivation in saltwater environments by utilizing abundant saltwater resources. The integrated aqua vegeculture system (iAVs) shows promise for water conservation and environmental resilience. Objective: To evaluate lettuce growth in iAVs with varying salt levels and investigate how saltwater impacts the biochemical properties and growth of both fish and lettuce. Methods: Over four months, iAVs were incorporated into an existing aquaponic system with lettuce (*Lactuca sativa*) and tilapia (*Oreochromis niloticus*). Four treatments were tested: freshwater (control-T1) and saltwater concentrations of 1 (T2—2.5%), 2 (T3—5%), and 3 (T4—7.5%). Results: Increased salinity did not affect the fish growth parameters, with a 90% survival rate across all groups. Higher salinity levels potentially improve fish farming efficiency. Lettuce growth was optimal in freshwater, with promising results at the T2 and T3 levels, but T4 had negative effects. Proximate composition analysis showed a decline in lettuce nutritional elements as the saltwater concentration increased. Conclusion: This approach could transform food production in coastal regions and arid nations, addressing food security and water scarcity issues while alleviating the pressure on freshwater sources.

## Simple Summary

Saline aquaponics integrate fish and crop cultivation in saltwater environments. The integrated aqua vegeculture system (iAVs) shows promise for water conservation. This study evaluated lettuce growth in iAVs with varying salt levels, and their impact on the yield and nutritional composition of fish and lettuce. The system tested lettuce (*Lactuca sativa*) and tilapia (*Oreochromis niloticus*) under four treatments: freshwater (control-T1) and saltwater concentrations of 1 (T2, 2.5%), 2 (T3, 5%), and 3 (T4, 7.5%). Fish showed 90% survival across all groups, with higher salinity potentially improving the farming efficiency. Lettuce growth was optimal in freshwater and viable at T2 and T3, but declined at T4. Nutritional elements in lettuce decreased at higher saltwater concentrations. This approach could transform food production in coastal and arid regions, while reducing freshwater usage.

## 1. Introduction

Urban agricultural systems such as greenhouse hydroponic production, aquaponics, sandponics, and integrative aquaculture systems are highly efficient options. These systems employ hydroponics, which eliminates the need for soil in agricultural farming. They also reduce freshwater consumption while increasing the crop yield per unit of land. Most importantly, they produce nutritious and diverse meals free from chemicals [1].

Saltwater aquaponics, also referred to as Mariponics, is an alternative to traditional aquaponics that combines saltwater for fish cultivation with salt-tolerant plants. This method is preferred because it enables the production of crops adapted to saline conditions, while simultaneously providing fresh seafood. Mariponic systems demonstrate higher productivity and reduced water consumption compared to conventional farming techniques [2]. In arid and semiarid regions, soil salinity poses a significant challenge to the agricultural output [3]. While offering similar advantages to freshwater aquaponics, saline aquaponics is particularly well suited for coastal, island, and estuarine settings, supporting the seafood industry. As freshwater resources have become increasingly limited worldwide [4], the abundance of saline water has made saltwater aquaponics an attractive solution. It is worth noting that only 2.5% of the world’s water is fresh, with the remaining 96.5% found in the oceans and seas [5].

Sandponics (SP) represents a different approach to plant cultivation. This method employs sand as the principal substrate for mechanical and biological filtration, as well as a growth medium for plants. SP offers a sustainable and eco-friendly production technique for a diverse range of crops, including vegetables, vines, and fruits. The ubiquity of sand in most regions facilitates widespread implementation of this method. Sand attributes, such as ease of sterilization, adaptability, recyclability, and cost-effectiveness compared to soil, enhance the efficiency, affordability, and risk reduction in SP technology [6]. However, SP faces several challenges, including the requirement for specialized operator training, potential nutrient deficiencies in crops due to inadequate fertilization, the need to identify suitable sand for cool-climate crops, and the high costs associated with heating systems [7]. Although SP alone may not comprehensively address food security concerns, they have the potential to foster thriving local crop production. This localized cultivation can contribute to improved nutrition in urban and peri-urban settings, ultimately leading to significant advancements in the development of more food-secure and nutritionally robust communities [8].

The integrated aqua-vegeculture (iAVs) system is an innovative and economical method that employs sand for filtration, biofiltration, and crop cultivation. The iAVs is an agricultural methodology that employs sand as a primary medium for mechanical filtration, biofiltration, and crop cultivation, with a critical focus on the biofilter volume to cult, Zure tank volume ratio for optimizing system function [9]. This approach offers benefits in terms of sustainability, accessibility, and affordability [9,10]. Despite some constraints, such as the need for specialized training and potential nutrient shortages, iAVs show potential for continuous organic crop production in controlled settings [11]. In the iAVs process, when the biofilter is filled with water during a pumping cycle, the removal of waste-containing aquaculture water ceases, allowing the biofilter to release purified water back into the tank [12].

The combination of aquaculture and olericulture offers several advantages. It enables efficient water resource conservation, allows the use of aquaculture waste products for food production and pollution reduction, facilitates intensive production of fish protein and vegetable crops, and results in lower operational costs than when using either system independently [13,14]. Freshwater and non-fish species, such as Nile tilapia (*O. niloticus*), which are recognized for their rapid growth and adaptability to various conditions, have demonstrated their capacity to reach marketable sizes in aquatic systems [15]. The ability of tilapia to adapt to different salinity levels makes them a suitable candidate for aquaculture in brackish and seawater environments [16]. The gradual acclimation of tilapia to saltwater significantly enhances their growth and survival rates [17]. In saltwater settings, tilapia may thrive with lower protein requirements for optimal growth, influenced by changes in salinity. Although the economic viability of seawater tilapia cultivation remains largely unexplored, cost-effective practices can be implemented with appropriate management. Agricultural researchers and planners are increasingly interested in utilizing diluted seawater for crop irrigation [18,19].

Lettuce, also known as *Lactuca sativa*, is a leafy vegetable that belongs to the Asteraceae family. It produces crisp leaves that are loosely arranged on the stalk and are commonly used in salads. Lettuce is valued for its dietary and medicinal properties [20,21]. It exhibits moderate salt tolerance, with salinity levels exceeding 2.0 and 2.6 dSm^−1^ affecting fresh yield and plant growth [22,23]. In the integrated aqua vegeculture system, plants use nutrients released from fish waste and the microbial breakdown of organic matter. Salinity can affect plant growth and seed germination, particularly in less salt-tolerant plants [24,25].

The growing demand for seafood products, especially in Middle Eastern countries where awareness of the health benefits of seafood has increased, has led to an increase in the use of saltwater aquaponic systems to cultivate highly salt-tolerant plants and seafood. This pilot study used the common Nile tilapia, *O. niloticus*, to compare the growth and proximate composition of lettuce (*L. sativa*) under various concentrations of aquaponic effluent saltwater in an integrated aquaculture and vegeculture system.

## 2. Materials and Methods

### 2.1. System Description

**Figure 1:**
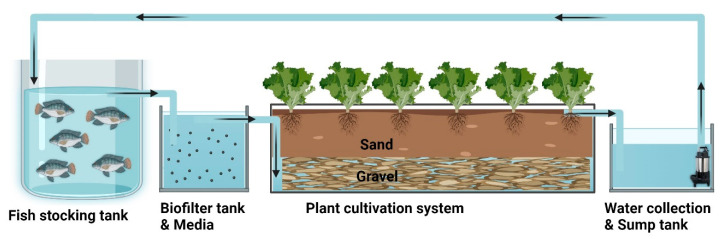
Diagram of the saltwater-based integrated aqua-vegeculture system (iAVs).

The experiment utilized the approach of establishing an integrated aquaculture and vegeculture system (iAVs), where the aquaculture water is directed to flow through a bed of growing material, such as gravel or sand [12,26]. This system comprises several components: a single fish tank, a biofilter tank, three plant culture troughs filled with gravel on the lower side, sand in the upper planting section, and a water collection sump tank. Additionally, a submersible aquarium pump (Atman AX-4000, Zhejiang, China) was used to recirculate the water back into the aquaculture tank. The model’s iAVs structure is shown in Figure 1, and the specific water volumes are listed in Table 1. The aquaponic system was maintained with continuous aeration (S53-AQ Sweetwater Regenerative Blower, Pentair Aquatic Eco-Systems, Inc., Apopka, FL, USA) to ensure sufficient oxygenation in both the aquaculture tank and plant cultivation units.

### 2.2. Procurement and Acclimatization of the Experimental Fish

The experimental fish, tilapia (*O. niloticus*), were sourced from the Tilapia Fish Breeding Center, Aquaculture Research Station, Falaj Hazza, UAE University. Selected fish of uniform size were directly introduced into the experimental system to facilitate acclimatization. After a one-week acclimatization period, the experimental setup underwent a gradual transition to include saltwater to conduct the salinity test experiment. Subsequently, the introduced fish were fed a diet consisting of commercial feed with 36% crude protein content procured from the ARASCO Feeds Corporation, Riyadh, Saudi Arabia.

### 2.3. Experimental Water Preparation

The experiment used a range of saltwater concentrations to investigate their effects on lettuce and fish growth and production. The freshwater serves as the control. Professional Sea Salt from Fauna Marin, Holzgerlingen, Germany, was used to create three distinct saltwater concentrations: 2.5%, 5%, and 7.5%. These concentrations were achieved by carefully diluting the sea salt with fresh water in the experimental tanks until the desired salinity level was reached. Throughout the experiment, the salinity was continuously monitored to ensure consistency and accuracy. To maintain the integrity of the experimental conditions, regular adjustments were made using artificial sea salt, as needed. This meticulous approach ensured that the desired salinity level remained constant throughout the study period. The salinity level was measured using a hand refractometer (ATAGO-S/Mill_-E_, Salinity 0 to 100%, Fukaya city, Japan). The total water volume and corresponding salinity levels for each experimental condition are presented in Table 2.

### 2.4. Plant Growth Setup

Seeds were purchased from a local agricultural market. To initiate lettuce growth, the seeds were germinated in pots filled with compost. After a germination period of 4–5 days, the seedlings were transferred to sand in the experimental system, maintaining a distance of 30 cm between each plant. As an additional measure, sticky sheets were placed around the plant cultivation zones to effectively trap and manage fly pests.

### 2.5. Fish Growth Parameters

Tilapia fish were exposed to different percentages of saline water (T1—0%, T2—2.5%, T3—5%, and T4—7.5%). Fish were separately placed in a 200 L plastic tank (20 fish per tank in replicate—20 × 2 = 40) for each treatment. Two replicates were used for each treatment. They were fed ad libitum with commercial feed twice a day. The salinity was gradually raised until the desired salinity levels were reached. Three fish were removed from each aquarium for blood sampling (six samples for each salinity treatment). The experiment was conducted over a period of four months. At the end of the experiment, various growth parameters of tilapia were measured, including weight gain (WG), specific growth rate (SGR), feed intake (FI), feed conversion ratio (FCR), and survival rate. These values were calculated using the following equations, previously used by Radhakrishnan et al. [27]:

(1) $$Survival\;rate\;\left(\%\right)=\frac{No\;of\;live\;fish\;(Final)}{Initial\;No\;of\;fish}$$

(2) $$Weight\;gain\;\left(g\right)=Final\;weight\;\left(g\right)-Initial\;weight\;(g)$$

(3) $$Specific\;growth\;rate\;\left(\%\right)=\frac{\log of\;final\;weight\;\left(g\right)-\log of\;initial\;weight\;(g)}{No\;of\;days}\times 100$$

(4) $$Feed\;conversion\;rate\;\left(\%\right)=\frac{Feed\;intake\;(g)}{Weight\;gain\;(g)}$$

The procedures for fish adaptation, acclimatization, euthanasia, and blood collection in the experiment adhered to the “ARRIVE” guidelines 2.0 [28]. Total length (to the nearest mm) and fresh weight (to the nearest mg) were measured, and blood samples (1 to 2 mL) were immediately taken from the caudal vein using vacutainer tubes (2 mL, without anticoagulant, BD brand) and needles (22G; 0.70 mm × 38 mm, BD brand). The samples were kept on ice before centrifugation for 5 min at 6000 rpm. Plasma was stored at −80 °C until analysis was performed. Plasma sodium, chloride, potassium, and glucose were analyzed in the blood using a chemistry analyzer (COBAS INTEGRA 400 plus, Roche Diagnostics, Mennheim, Germany). Muscle tissue samples (1–2 g) without the skin were weighed to the nearest milligram immediately after removal. They were dried at 80 °C for 24 h and reweighed every 24 h until a constant weight was obtained. The total liver was removed and weighed to determine the hepatosomatic index. The muscle water content and hematosomatic index have been verified [29,30].

(5) $$Muscle\;moisture\;\left(\%\right)=\frac{\left(Wet\;weight-Dry\;weight\;\right)}{Wet\;weight}\times 100$$

(6) $$Hepatosomatic\;index\;HSI\;\left(\%\right)=\frac{Wet\;liver\;weight}{Body\;weight}\times 100$$

### 2.6. Plant Growth Parameters

During the experiment, lettuce was harvested at regular intervals every 30 d, and a new seed was planted to initiate a new crop cycle. The harvested lettuce was assessed and characterized using various parameters, including measurements of length (shoot and root length), total weight, head weight, leaf weight, leaf length, leaf width, and average number of leaves. These measurements allowed for a comprehensive evaluation of lettuce characteristics at each harvest stage, providing valuable insights into the growth and development of the crop throughout the experiment.

The sludge management during the experiment involved a systematic process. Sludge was collected daily by siphoning method, and any floating sludge was removed.

### 2.7. Water Quality Parameters

Water quality analyses were performed weekly in fish tanks. These included pH, temperature, electrical conductivity, and total dissolved solids (TDS), which were measured using a portable meter (CyberScan, PC 300 Series, Thermo Fisher Scientific, Eutech Instruments, Singapore). The salinity level was analyzed by the hand Refractometer (ATAGO-S/Mill_-E,_ ATAGO Co., Ltd., Fukaya City, Japan). The dissolved oxygen (DO), Total Ammonia Nitrogen (TAN), nitrite, and nitrate were measured using a HACH DR900 multiparameter calorimeter (Lenntech, HACH company, Loveland, CO, USA).

### 2.8. Proximate Composition Analysis

The experimental fish and lettuce samples were analyzed in triplicate for their proximate composition. Moisture content was determined using a forced air oven, crude protein using the macro-Kjeldahl method, crude fat through Ether extraction, and total ash using a muffle furnace (at 550 °C) for 24 h. Biochemical analysis (dry matter, moisture, crude protein, crude lipid, fiber, and ash of carcass) was determined by using standard procedures of [31]. The fish carcass total carbohydrates were estimated by the following subtraction method (% of carbohydrates = 100 − %moisture − %protein − %lipid − %mineral). The macro- and micro-mineral contents were analyzed using an ICP-MS instrument (NexION 300X, PerkinElmer, Springfield, IL, USA), following the method described in [32].

### 2.9. Experimental Sampling and Statistical Analysis

The experiment was conducted in triplicate on different independent aquaponic systems over a four-month period. Lettuce was harvested three times during the four-month period, with each harvest requiring 30–35 days. For each harvest, three random lettuce samples were selected for analysis. Growth and biochemical analyses were performed monthly, and statistical analyses were conducted using average monthly data. Water quality parameters were measured twice a month, and the average values were used for statistical analysis. Fish growth parameters were recorded on the initial and final days of the experiment and included in the statistical analysis.

The experimental design was completely randomized (CRD), and each treatment was replicated three times. All data were subjected to one-way ANOVA to determine significant (*p* < 0.05) differences among the treatment means. All statistical analyses were performed using the IBM SPSS Statistics software, Version: 29.0.0.0 (241), IBM, Chicago, IL, USA.

## 3. Results 

### 3.1. Water Quality Parameters

During this study, the water quality parameters of the saline aquaponic effluent samples were recorded, and the results are provided in Table 3 for each month.

i. Temperature: Across all experimental conditions, the temperature remained relatively consistent, ranging from approximately 21 to 22.7 °C. This stability suggests that saltwater inclusion levels do not significantly influence water temperature.

ii. Dissolved Oxygen (DO): The dissolved oxygen levels showed variations but generally remained within acceptable ranges for aquaponics systems. The values ranged from 5.91 mg/L to 7.63 mg/L, indicating adequate oxygen availability for the aquatic organisms.

iii. pH: The pH values exhibited some variability but generally fell within the neutral to slightly alkaline range (6.20 to 7.10). This suggests that saltwater inclusion does not cause significant alterations in the acidity of the system, thereby maintaining a suitable pH for aquaponics.

iv. Total Dissolved Solids (TDSs) and Electrical Conductivity (EC): TDS and EC values demonstrated substantial differences across the experimental conditions. Higher saltwater concentrations resulted in elevated TDS and EC values, indicating an increase in the concentration of dissolved substances and the overall conductivity of the water.

v. Ammonia, Nitrate, and Nitrite Concentrations: Ammonia levels are generally low across all experiments, ranging from 0.12 mg/L to 0.81 mg/L. These data suggest that the inclusion of saltwater did not lead to a significant increase in ammonia concentration. The nitrate and nitrite concentrations also varied, with higher values observed in experiments with increased saltwater concentrations.

### 3.2. Fish Growth Parameters

The experimental results presented in Table 4 highlight the growth and nutritional indices of fish subjected to the four different treatment groups (T1, T2, T3, and T4). Statistical analysis revealed significant differences in final length and weight among the groups. Specifically, T3 and T4 significantly increased the lengths (12.33 ± 0.58 cm and 12.67 ± 0.58 cm, respectively) compared to T1 and T2, indicating enhanced growth in these treatments (*p* < 0.05). However, final weights displayed a reverse trend, with T1 and T2 showing higher values (183.33 ± 7.64 g and 186.00 ± 8.54 g, respectively), suggesting better weight retention in these groups. The study examined length gain in fish across four treatment groups (T1–T4). Initially, the fish lengths ranged from 3.87 ± 0.35 to 4.50 ± 0.50. After the experimental period, the final lengths increased significantly, ranging from 11.97 ± 0.58 to 12.67 ± 1.00. Interestingly, there were no statistically significant differences in final lengths among the treatment groups, as indicated by the same superscript letter ‘a’ for all treatments. Weight gain followed a similar pattern, with T1 and T2 significantly outperforming T4 (*p* < 0.05). Feed intake and feed conversion ratio showed no significant differences across treatments, implying a consistent feeding efficiency. The survival rates remained high and uniform (≥90%), indicating robust experimental conditions. Notably, the hepatosomatic index was significantly reduced in T4 (0.83 ± 0.06%), potentially indicating little stress or physiological adaptation under this treatment.

### 3.3. Proximate Composition of the Fish Carcass and Blood Chemistry

Analysis of fish muscle proximate composition and serum biochemical parameters (Table 5) revealed significant trends under different salinity treatments (T1 to T4). The muscle proximate composition showed no significant differences in moisture, ash, crude protein, fat, and fiber content among treatments, indicating stable nutrient retention across varying salinities (*p* > 0.05). However, carbohydrate content varied significantly, with T1 and T3 showing higher values (3.74 ± 0.34% and 3.68 ± 0.20%, respectively) than T2 and T4 (*p* < 0.05), suggesting differential carbohydrate metabolism under these conditions.

For serum biochemistry, sodium, and chloride levels increased significantly with salinity, peaking at T3 (50.09 ± 1.73 mM for sodium and 34.47 ± 1.48 mM for chloride) and T4 (49.50 ± 6.01 mM for sodium and 41.26 ± 1.27 mM for chloride), reflecting salinity-induced ionic regulation (*p* < 0.05). Potassium and glucose levels showed no significant differences across treatments (*p* > 0.05), indicating a consistent physiological response to these parameters. These findings highlight the influence of salinity on specific biochemical adaptations in fish, with potential implications for optimizing aquaculture practices under variable salinity conditions.

### 3.4. Lettuce Plant Growth Parameters and Yield

In the present study, the growth parameters of lettuce grown in different saltwater cultivation systems were measured, and the final average production results were displayed. The monthly averages are presented in Table 6.

i. Shoot to Root Length (cm): A clear decreasing trend in length was observed with increasing salinity. The control group and the 2.5% saltwater group showed similar lengths, whereas the 5% and 7.5% saltwater groups exhibited significant reductions, indicating a negative correlation between salinity and overall plant length.

ii. Shoot Length (cm) and Root Length (cm): Shoot and root lengths followed a similar pattern of decline at higher salinity levels. This reduction was more pronounced in root length, emphasizing the sensitivity of the root system to increased salinity.

iii. Total Weight (g): A substantial decrease in total plant weight was evident as the salinity increased. The control group had the highest total weight, whereas the 7.5% saltwater group showed a significant reduction, emphasizing the adverse impact of elevated salinity on the overall plant weight.

iv. Shoot Weight (Head) (g) and Root Weight (g): Both shoot and root weights exhibited consistent decline with increasing salinity. The reduction in shoot weight, particularly head weight, suggests compromised growth of the marketable part of the plant.

v. Leaf Weight (g), Length (cm), Width (cm), and Average Number of Leaves: Salinity levels negatively affected leaf characteristics. Leaf weight, length, and width consistently decreased with increasing salinity, indicating stress to leaf development. The average number of leaves also decreased significantly, further emphasizing the negative impact on overall leaf production.

### 3.5. Proximate Composition Analysis

The results of the proximate composition of lettuce leaves are presented in Table 7.

i. Moisture Content: Lettuce maintained a high moisture content under low salinity (2.5%) but showed a significant reduction under high salinity (7.5%). High salinity affects the water retention capability of lettuce, thereby affecting its texture and freshness.

ii. Protein (CP) and Fat Content: The crude protein (CP) content showed a decreasing trend with increasing salinity. The protein content was highest in the control group, measuring approximately 28.70 mg/kg. When exposed to 2.5% saltwater, the protein content decreased slightly to approximately 24.23 mg/kg. This decline continued with 5% saltwater, where the protein content decreased further to approximately 20.90 mg/kg. At 7.5% salinity, the protein content was significantly reduced to approximately 14.63 mg/kg.

The fat content was similar for the control group and the 2.5% saltwater treatment at approximately 2.83 mg/kg and 2.93 mg/kg, respectively. A slight decrease in fat content was observed at 5% salinity, which decreased to approximately 2.53 mg/kg. At 7.5% salinity, the fat content was significantly reduced to approximately 1.37 mg/kg.

iii. Fiber (CF) and Ash Content: Fiber content of lettuce exhibited a slight increase at 2.5% salinity compared with that of the control. The 2.5% saltwater group exhibited the highest fiber concentration (12.53 mg/kg), whereas the 7.5% saltwater treatment had the lowest (8.27 mg/kg).

The ash content also followed a similar trend, with the 2.5% group showing the highest ash concentration (20.77%), which was similar to that of the control group (20.53%), while the 7.5% saltwater treatment displayed the lowest (9.63%). These trends indicate that higher salinity levels may contribute to a reduction in the fiber and ash content in lettuce leaves.

iv. Nitrogen Free Extract (NFE): The NFE demonstrated a decreasing trend with increasing salinity levels. The nitrogen-free extract content was the highest in the control group at approximately 33.33%. For both the 2.5% and 5% saltwater treatments, the NFE content remained relatively stable at approximately 31.40% and 32.57%, respectively. However, at 7.5% salinity, there was a significant decrease in NFE content to approximately 22.70%, suggesting that lettuce may allocate fewer resources for non-fiber carbohydrate accumulation under elevated salinity conditions.

v. Chlorophyll Content: Chlorophyll content exhibited a decreasing trend with increasing salinity. The chlorophyll content was highest in the control group, measuring approximately 1.40 mg/kg. For the 2.5% and 5% saltwater treatments, the chlorophyll content was similar, at approximately 0.95 mg/kg and 0.94 mg/kg, respectively. A significant decrease in chlorophyll content was observed at 7.5% salinity, dropping to approximately 0.82 mg/kg. This observation aligns with the known effects of salinity on chlorophyll synthesis, indicating a potential reduction in photosynthetic activity under high salinity conditions.

#### Macro and Micro Elements Concentration of Lettuce

In this study, the control and various salinity levels of cultivated lettuce leaves were examined for macro- and micro-mineral concentrations. Table 8 presents the results of the study.

i. Macro minerals: This study investigated the effect of varying salinity levels (control, 2.5%, 5%, and 7.5% saltwater) on macro-element concentrations in cultivated lettuce. The results showed that calcium, potassium, magnesium, phosphorus, and sulfur concentrations generally decreased with increasing salinity. Specifically, calcium and magnesium levels exhibited significant decreases, whereas potassium and phosphorus levels showed moderate decreases. Sulfur levels remained relatively stable at lower salinities but decreased noticeably at higher levels. Sodium levels increased with increasing salinity concentrations. The 7.5% saltwater treatment exhibited the highest sodium content, indicating a direct relationship between sodium availability in the water and its accumulation in lettuce tissues. These findings suggest that although lettuce can tolerate moderate salinity (up to 2.5%), high salinity adversely affects the uptake of essential macro-elements crucial for plant growth and nutrition. Further investigation is required to understand the specific interactions that influence mineral uptake in lettuce under different salinity conditions.

ii. Trace Elements (Co, Fe, Mn, and Zn): This study also examined the effect of salinity on trace element concentrations (Co, Fe, Mn, and Zn) in lettuce leaves. The iron (Fe) content in lettuce showed a slight increase from 144.03 ppm in the control group to 148.40 ppm in the 2.5% saltwater treatment. The concentrations of other trace elements such as Co, Mn, and Zn showed a significant decline with increasing salinity. However, the concentrations of calcium (Ca), potassium (K), magnesium (Mg), phosphorus (P), and sulfur (S) were similar between the control and 2.5% saltwater treatments. This similarity supports the argument for promoting low salinity conditions (2.5% salinity) for lettuce cultivation, as it minimally affects mineral composition compared to higher salinity treatments.

## 4. Discussion

### 4.1. Water Quality Parameters

These results demonstrated the influence of saltwater inclusion on key physicochemical parameters, notably TDS, EC, and nitrogen compounds. However, crucial factors such as temperature, DO, and pH maintain stability within acceptable ranges for aquaponics, indicating resilience of the system to saltwater inclusion.

Plants, particularly lettuce, thrive in the pH range of 5.5–6.5, emphasizing the importance of maintaining optimal pH levels for nutrient absorption [33,34]. This study affirms that, despite saltwater inclusion, the iAVs sustained favorable conditions for both fish and plant growth.

The water quality parameters remained satisfactory in systems with up to 7.5% saltwater, with a minimal impact on nitrification levels. However, this study acknowledges the need for further research to explore potential long-term effects on nitrification and overall system stability.

Referring to Yap and Teo [21], elevated sodium ion concentrations resulting from saltwater utilization adversely affected lettuce growth, potentially causing nutrient deprivation and increasing susceptibility to root diseases. These results align with Merrill et al. [35], highlighting deficiencies in crucial nutrients due to the elevated potassium content in saltwater.

The increased Ca^2+^ concentration in saltwater leads to the sequestration of essential elements, affecting nutrient absorption by plants [36,37]. This study concurs with Arora et al. [38] regarding the impact of cationic nutrients, including phosphorus, boron, copper, and zinc.

Although chloride competition with nitrate is noted [39], an increased chloride concentration hinders nitrate uptake, potentially affecting plant growth [40]. High chloride levels can lead to ion toxicity and can inhibit plant development [41]. This emphasizes the limited salinity tolerance of lettuce, suggesting that the iAVs is advantageous for lettuce cultivation.

### 4.2. Fish Growth Parameters, Proximate Composition, and Blood Chemistry

Fish growth in terms of weight was not adversely affected by salinity levels. Fish in all salinity-treatment groups gained weight. The 2.5% and 5% saltwater groups showed the highest final weights and weight gains, indicating that moderate salinity levels might slightly enhance the weight gain in fish. Fish demonstrated high survival rates across all salinity levels, indicating resilience and adaptability.

The feed consumption of the fish remained stable across environments with different salinities. FCR exhibited minor variations across different salinity levels. A lower FCR indicates more efficient feed conversion to body weight [42]. The 2.5% saltwater condition showed the most efficient feed conversion, whereas the 7.5% saltwater condition showed a slightly lower efficiency. However, these differences were minor, suggesting that overall feed efficiency was relatively stable across varying salinity levels. The 7.5% saltwater group displayed a slightly lower weight gain and FCR, suggesting a mild negative impact at higher salinity levels. The favorable effects of moderate salinity on fish growth parameters may be linked to improved osmoregulation [43]. Vieira et al. [44] also reported that Nile tilapia could be reared in water salinities up to 7 g L^−1^ without damaging certain parameters. This study implies that salinity is a key factor that affects fish growth [45]. These results indicate that careful consideration of salinity levels in aquaponic-based iAVs can enhance fish farming efficiency and foster sustainable and resilient aquaculture practices [16].

Researchers have explored various strategies to optimize the welfare and productivity of farmed tilapia. One area of focus has been the biochemical composition and salt tolerance of these fish. Studies have shown that tilapia can adapt to a range of salinities, with changes in their carcass composition and liver size (hepatosomatic index) reflecting their physiological responses [46]. Understanding these biochemical adaptations is crucial for developing effective management practices to enhance the growth and health of tilapia in aquaculture. Indeed, tilapia inhabits a diverse array of aquatic habitats, from freshwater to brackish environments, and even hot springs and highly alkaline waters [47]. The ability of tilapia to tolerate a wide range of salinity conditions is attributable to their robust osmoregulatory mechanisms, which enable them to maintain their internal fluid balance by actively regulating the exchange of water and ions with the external environment [46,47]. When exposed to low salinity conditions, tilapia can experience physiological changes that affect their body composition and organ size [48].

Earlier findings indicated that tilapia, such as Nile tilapia and Mozambique tilapia, exhibited remarkable salinity tolerance, with the ability to acclimate to varying salinity levels [48,49,50]. For instance, the Mozambique tilapia has been shown to possess excellent salinity tolerance, which is attributed to its capacity to develop specialized chloride cells in the gill epithelium and opercular membrane in response to increased environmental salinity [50]. Similarly, studies on Nile tilapia have revealed that these fish can adapt to a range of salinity conditions, with physiological and ionic changes observed in response to low and high salinity stressors [48]. These adaptations include changes in the protein content of the carcass and the size of the liver, which can serve as indicators of the fish’s overall health and physiological status [51].

These findings on the serum biochemistry of tilapia exposed to different salinity levels provided valuable insights into their physiological adaptations. The significant increases in sodium and chloride levels at higher salinity treatments (T3 and T4) reflect the capacity of fish to actively regulate their ionic balance in response to external salinity conditions. This is a hallmark of the excellent salinity tolerance exhibited by tilapia, which can be attributed to their ability to develop specialized chloride cells in the gill epithelium and opercular membrane [50]. In addition, the increased hepatosomatic index observed in fish reared under elevated salinity conditions suggests that the liver plays a crucial role in osmoregulation, likely because of its involvement in the production of osmotically active substances and excretion of excess ions [52].

In contrast, the lack of significant differences in potassium and glucose levels across the salinity treatments suggests a consistent physiological response in these parameters, despite varying salinities. This indicates that fish can maintain homeostasis in certain blood biochemical markers, even as they undergo osmoregulatory adjustments to cope with the changing salinity [50,53].

These findings underscore the complex and adaptive nature of the physiological responses of tilapia to salinity stress. Understanding these biochemical adaptations is crucial for developing effective management practices in aquaculture, as they can help optimize the growth and health of tilapia under variable salinity conditions.

### 4.3. Growth Performance and Biochemical Quality of the Lettuce

In the present study, lettuce exhibited normal growth in freshwater; however, growth parameters declined with increasing levels of saltwater inclusion. Notably, the 2.5% and 5% inclusion levels demonstrated superior growth compared with the 7.5% saltwater inclusion. These findings suggest that lettuce plants are sensitive to elevated salinity in iAVs but can tolerate some degree of salinity. Higher salinity negatively affected various growth parameters, including the overall plant length, shoot and root lengths, and weight distribution. Decreased shoot and root lengths, coupled with reduced shoot weight, may indicate challenges in water and nutrient uptake, leading to compromised plant development [54].

Yap and Teo [21] reported the inability of lettuce to thrive in saltwater aquaponic systems, emphasizing the critical role of maintaining a low salt environment for optimal growth and productivity. Similarly, Turhan et al. [23] found in their study that the inclusion of modest amounts of seawater in the irrigation water had no significant effect on lettuce yield or quality. Maintaining low salt levels with a recyclable nutrient solution in irrigation water is imperative for achieving the best crop yield [54].

Further research is necessary to comprehend the long-term effects of saltwater irrigation on crop yield and quality and to explore potential mitigation strategies to minimize these adverse impacts. Some studies have indicated that certain crops can tolerate higher salinity levels and maintain acceptable yields, whereas others are more sensitive and experience significant reductions in productivity [54,55,56]. Therefore, a comprehensive understanding of these intricate interactions is crucial for developing effective strategies to manage salinity and optimize crop production in saline environments [36].

### 4.4. Proximate Composition Analysis

The consistent moisture content across the different salinity treatments suggests that lettuce maintained its hydration status under diverse saline conditions. This stability is crucial for understanding water management in lettuce cultivation, indicating that the plant can regulate water uptake and retention even in the presence of elevated salinity. The decrease in protein and fat contents with increasing salinity implies a potential trade-off between nutrient accumulation and saline stress. As salinity increases, lettuce may allocate fewer resources to protein and fat synthesis, affecting the nutritional quality of the harvested leaves [57,58,59,60,61]. This finding prompted further exploration of the molecular mechanisms governing nutrient metabolism under saline conditions. The diminishing trends in fiber and ash content suggest that higher salinity levels may contribute to reduced structural components and mineral content in lettuce leaves [62,63,64]. This has implications for the dietary fiber and mineral contents of harvested produce. Understanding these dynamics is vital for consumers and nutritionists seeking the specific nutritional benefits of lettuce. The decline in non-fiber carbohydrate content under elevated salinity conditions indicated alterations in carbohydrate allocation within the plant [65,66,67]. This observation has implications for energy storage and utilization, potentially influencing the overall calorie content of lettuce leaves [60,68]. Further research is required to elucidate the underlying physiological mechanisms governing carbohydrate metabolism in response to saline stress [69].

The reduction in chlorophyll content at higher salinity levels aligns with the known effects of salinity stress on photosynthetic activity [70,71]. Decreased chlorophyll levels indicate potential limitations in the ability of plants to harness light energy for photosynthesis [72]. This finding underscores the importance of optimizing salinity to maintain robust photosynthetic processes that are crucial for overall plant growth and productivity [73,74,75]. The collective effects of salinity on various biochemical components underscore the need for a nuanced approach to lettuce cultivation under saline conditions. Although plants exhibit resilience in maintaining moisture content, the observed reductions in protein, fat, fiber, ash, NFE, and chlorophyll content signal potential challenges in achieving optimal nutritional outcomes. These findings suggest that excessive saltwater irrigation can decrease the overall nutritional value of lettuce, which has implications for human consumption.

### 4.5. Macro and Micro Elements Concentration of Lettuce

The observed increase in calcium and magnesium concentrations with increasing salinity suggests a positive correlation between salinity and the uptake of these essential minerals. These findings align with those of previous studies, indicating that certain plants exhibit enhanced calcium and magnesium absorption in saline environments [76]. The elevated sodium content in lettuce leaves under high-salinity conditions underscores the ability of the plant to accumulate this element. Sodium, although not traditionally considered a nutrient, plays a role in osmoregulation and may contribute to the adaptation of plants to saline conditions [58,60,76,77]. Phosphorus and sulfur concentrations remain relatively stable across all treatments, indicating robust nutrient homeostasis [17]. This stability suggests that, within the tested salinity range, lettuce plants maintain consistent levels of these essential elements, which are crucial for various physiological processes. The responses of trace elements, such as iron, manganese, copper, molybdenum, and zinc, are diverse, indicating the complexity of nutrient interactions in saline environments. The 2.5% saltwater treatment resulted in elevated concentrations of certain trace elements, suggesting an intricate relationship between salinity and trace mineral uptake. The ability of lettuce plants to adapt to a range of salinity levels is evident from their capacity to take up and accumulate minerals under different conditions [78,79,80]. This adaptability is crucial for aquaponic systems where fluctuations in water salinity may occur. The observed trends in mineral composition have significant implications for lettuce cultivation. Growers can use the effects of salinity on nutrient uptake to modulate the nutritional content of lettuce leaves. Careful management of salinity can be employed to enhance the nutritional profile of cultivated crops.

## 5. Conclusions

In conclusion, this study investigated the effects of varying salinity levels on both fish and lettuce in an integrated aquaculture system (iAVs). The results revealed that mild increases in salinity of up to 5% saltwater did not adversely affect fish growth parameters, demonstrating resilience and adaptability. However, higher salinity levels, particularly 7.5%, had a mild negative impact on fish performance. In contrast, lettuce displayed sensitivity to increased salinity, with significant reductions in various growth parameters and biochemical parameters. The study specifically noted that a 2.5% saltwater concentration in irrigation did not significantly affect the fresh yield or quality of lettuce crops. The collective findings emphasize the need for a careful and balanced approach to managing salinity levels in iAVs. Although moderate salinity may enhance fish farming efficiency, it poses challenges for optimal lettuce cultivation, thus impacting nutritional outcomes. Further research is recommended to explore the long-term effects, mitigation strategies, and potential benefits of nutrient modulation to optimize crop production in saline environments.

## Figures and Tables

**Table 1:** Experimental system water volume.

Part of the System	Height (m)	Length/Width (m)	Filling Depth (m)	Water Volume (m^3^)	No of the Tanks	Total Water Volume (m^3^)
Fish stocking tank	0.900	0.548	0.838	0.198	2	0.396
Biofilter tank	0.900	0.548	0.838	0.198	2	0.396
Plant culture raceway	0.277	0.900	0.237	0.050	3	0.150
Collect and pumping tank	0.577	0.900	0.347	0.143	1	0.143
Total						1.085

**Table 2:** Experimental saltwater preparation.

Experiment	Total Water Volume (m^3^)	Average Salinity (%)
(T1) Control	1.085	0
(T2)	1.085	2.5
(T3)	1.085	5
(T4)	1.085	7.5

**Table 3:** Physicochemical parameters of the iAVs experimental water.

Experiment	Months	Temperature (°C)	Dissolved Oxygen (mg/L)	pH	Total Dissolved Solids (ppm)	Electrical Conductivity (S/m)	Ammonia (mg/L)	Nitrate (mg/L)	Nitrite (mg/L)
T1	1	22.16 ± 1.02 ^a^	7.08 ± 0.77 ^a^	6.81 ± 0.19 ^ab^	377.38 ± 45.08 ^de^	0.15 ± 0.07 ^c^	0.25 ± 0.18 ^e^	6.96 ± 1.79 ^a^	0.23 ± 0.11 ^ab^
	2	22.79 ± 1.14 ^a^	5.96 ± 0.34 ^b^	6.26 ± 0.05 ^b^	665.08 ± 55.27 ^bc^	0.33 ± 0.08 ^b^	0.61 ± 0.08 ^abc^	13.45 ± 0.71 ^b^	0.18 ± 0.01 ^ab^
	3	22.65 ± 1.05 ^a^	5.76 ± 0.35 ^b^	6.86 ± 0.56 ^ab^	848.40 ± 85.42 ^ab^	0.45 ± 0.06 ^a^	0.81 ± 0.07 ^a^	15.67 ± 0.64 ^b^	0.29 ± 0.02 ^ab^
T2	1	21.56 ± 1.46 ^a^	6.70 ± 1.19 ^a^	6.89 ± 0.53 ^ab^	382.67 ± 83.91 ^de^	0.26 ± 0.11 ^c^	0.23 ± 0.17 ^e^	5.55 ± 2.02 ^a^	0.13 ± 0.06 ^b^
	2	22.26 ± 1.35 ^a^	6.21 ± 0.35 ^b^	6.20 ± 0.03 ^b^	679.15 ± 198.1 ^bc^	0.49 ± 0.07 ^ab^	0.49 ± 0.04 ^cd^	14.94 ± 2.01 ^b^	0.26 ± 0.04 ^ab^
	3	21.94 ± 1.56 ^a^	6.22 ± 0.53 ^b^	6.60 ± 0.11 ^ab^	991.63 ± 6.79 ^a^	0.53 ± 0.07 ^a^	0.73 ± 0.05 ^ab^	15.30 ± 0.29 ^b^	0.46 ± 0.27 ^a^
T3	1	21.69 ± 1.33 ^a^	7.48 ± 0.62 ^a^	6.68 ± 0.12 ^ab^	350.05 ± 20.34 ^de^	0.29 ± 0.03 ^c^	0.30 ± 0.11 ^de^	6.40 ± 0.95 ^a^	0.19 ± 0.15 ^ab^
	2	22.17 ± 1.15 ^a^	5.85 ± 0.52 ^bc^	6.24 ± 0.01 ^b^	575.56 ± 65.44 ^cd^	0.51 ± 0.04 ^b^	0.58 ± 0.02 ^bc^	14.36 ± 4.12 ^b^	0.23 ± 0.08 ^ab^
	3	22.25 ± 1.39 ^a^	5.55 ± 0.71 ^bc^	7.06 ± 0.18 ^a^	671.33 ± 7.78 ^bc^	0.65 ± 0.10 ^a^	0.83 ± 0.08 ^a^	15.42 ± 1.07 ^b^	0.29 ± 0.01 ^ab^
T4	1	22.11 ± 1.21 ^a^	7.35 ± 0.59 ^a^	7.10 ± 0.22 ^a^	334.42 ± 15.68 ^de^	0.30 ± 0.03 ^c^	0.15 ± 0.04 ^e^	4.91 ± 1.12 ^a^	0.12 ± 0.05 ^b^
	2	21.46 ± 0.29 ^a^	6.38 ± 0.79 ^b^	6.63 ± 0.06 ^ab^	386.84 ± 31.69 ^de^	0.57 ± 0.11 ^b^	0.53 ± 0.08 ^bc^	7.65 ± 0.82 ^b^	0.30 ± 0.01 ^ab^
	3	22.15 ± 1.21 ^a^	4.77 ± 0.89 ^c^	6.85 ± 0.47 ^ab^	836.63 ± 225.99 ^ab^	0.76 ± 0.09 ^a^	0.73 ± 0.06 ^ab^	14.88 ± 0.30 ^b^	0.47 ± 0.25 ^a^

Each value is Mean ± SD of 2 individual observations. Means in the same column sharing the same superscript letter are not significantly different and determined by DMRT test (*p* < 0.05).

**Table 4:** Fish growth parameters and nutritional indices of the experimental fish.

Growth Parameters	T1	T2	T3	T4
No. of Fish (Initial)	20.00 ± 0.00	20.00 ± 0.00	20.00 ± 0.00	20.00 ± 0.00
Length Initial	4.13 ± 0.32	4.47 ± 0.55	3.87 ± 0.35	4.50 ± 0.50
Weight Initial	13.67 ± 0.29	14.13 ± 0.60	13.83 ± 0.29	14.07 ± 0.51
No of Fish Final	18.00 ± 1.00 ^a^	18.00 ± 1.00 ^a^	18.33 ± 0.58 ^a^	18.00 ± 1.00 ^a^
Length Final	11.97 ± 0.58 ^a^	12.67 ± 1.00 ^a^	12.33 ± 0.58 ^a^	12.27 ± 0.58 ^a^
Weight Final	183.33 ± 7.64 ^a^	186.00 ± 8.54 ^a^	178.33 ± 7.64 ^a^	166.67 ± 7.64 ^b^
Weight gain (g)	169.67 ± 7.42 ^a^	171.87 ± 8.26 ^a^	164.50 ± 7.70 ^a^	152.60 ± 7.38 ^b^
Feed intake (g)	147.33 ± 2.52 ^a^	149.67 ± 4.51 ^a^	143.67 ± 2.08 ^a^	145.67 ± 3.06 ^a^
Feed conversion ratio (%)	0.80 ± 0.05 ^a^	0.81 ± 0.05 ^a^	0.81 ± 0.03 ^a^	0.82 ± 0.05 ^a^
Survival (%)	90.00 ± 5.00 ^a^	90.00 ± 5.00 ^a^	91.67 ± 2.89 ^a^	90.00 ± 5.00 ^a^
Hepatosomatic index (%)	1.09 ± 0.04 ^a^	1.00 ± 0.05 ^a^	1.00 ± 0.03 ^a^	1.05 ± 0.07 ^a^
Specific growth rate (%)	4.04 ± 0.35 ^a^	4.09 ± 0.38 ^a^	3.99 ± 0.26 ^a^	3.63 ± 0.37 ^a^

Each value is Mean ± SD of the 2 individual observations. Means in the same row sharing the same superscript letter are not significantly different and determined by DMRT test (*p* < 0.05).

**Table 5:** Fish muscle proximate composition and serum biochemistry.

Carcass Proximate Composition (%)	T1	T2	T3	T4
Moisture	80.00 ± 0.64 ^a^	79.71 ± 1.77 ^a^	79.78 ± 1.77 ^a^	80.05 ± 0.28 ^a^
Ash	1.60 ± 0.35 ^a^	1.53 ± 0.15 ^a^	1.47 ± 0.07 ^a^	1.26 ± 0.15 ^a^
Crude protein	14.75 ± 0.78 ^a^	15.47 ± 0.35 ^a^	15.32 ± 0.66 ^a^	15.09 ± 1.10 ^a^
Fat	2.40 ± 0.49 ^a^	2.43 ± 0.15 ^a^	2.37 ± 0.15 ^a^	2.67 ± 0.32 ^a^
Fiber	1.50 ± 0.09 ^a^	1.49 ± 0.08 ^a^	1.50 ± 0.05 ^a^	1.49 ± 0.22 ^a^
Carbohydrate	3.74 ± 0.34 ^a^	3.09 ± 0.11 ^bc^	3.68 ± 0.20 ^ab^	2.98 ± 0.18 ^c^
Serum biochemical (mM)	T1	T2	T3	T4
Sodium	26.75 ± 2.19 ^c^	37.90 ± 1.73 ^b^	50.09 ± 1.73 ^a^	49.50 ± 6.01 ^a^
Chloride	23.60 ± 1.06 ^d^	30.53 ± 1.27 ^c^	34.47 ± 1.48 ^b^	41.26 ± 1.27 ^a^
Potassium	3.90 ± 0.95 ^a^	4.32 ± 0.12 ^a^	3.48 ± 0.12 ^a^	4.04 ± 0.25 ^a^
Glucose	5.40 ± 0.49 ^a^	5.43 ± 0.15 ^a^	4.87 ± 0.56 ^a^	4.67 ± 0.32 ^a^

Each value is Mean ± SD of the 2 individual observations. Means in the same row sharing the same superscript letter are not significantly different and determined by DMRT test (*p* < 0.05).

**Table 6:** Monthly average growth parameter of the iAVs cultivated lettuce plant.

Experiment	Harvest	TL	GL	RL	TW	GW	RW	LW	LL	LWI	LN
T1	1	84.80 ± 1.20 ^a^	33.35 ± 2.12 ^a^	51.45 ± 0.92 ^ab^	548.39 ± 35.42 ^a^	473.18 ± 39.66 ^a^	75.21 ± 4.24 ^a^	34.51 ± 1.32 ^a^	31.19 ± 0.83 ^a^	27.88 ± 0.81 ^a^	20.00 ± 1.41 ^a^
	2	75.70 ± 1.41 ^b^	27.15 ± 1.27 ^b^	48.55 ± 0.14 ^ab^	550.48 ± 7.07 ^a^	479.91 ± 5.34 ^a^	70.57 ± 1.73 ^b^	26.83 ± 1.32 ^b^	26.87 ± 0.83 ^b^	24.38 ± 0.81 ^b^	18.00 ± 1.41 ^bc^
	3	66.30 ± 3.54 ^c^	26.30 ± 0.71 ^bc^	40.00 ± 2.83 ^cd^	402.57 ± 14.68 ^c^	342.78 ± 15.38 ^d^	59.79 ± 0.71 ^c^	21.96 ± 1.32 ^bc^	25.64 ± 0.83 ^bc^	22.98 ± 0.81 ^b^	16.00 ± 1.41 ^bc^
T2	1	86.30 ± 1.41 ^a^	31.30 ± 0.71 ^a^	55.00 ± 2.2 ^a^	489.84 ± 8.29 ^b^	437.33 ± 11.12 ^b^	52.51 ± 2.83 ^b^	34.67 ± 1.32 ^a^	30.99 ± 0.83 ^a^	27.68 ± 0.81 ^a^	21.00 ± 2.83 ^a^
	2	74.10 ± 1.41 ^b^	27.20 ± 0.71 ^b^	46.90 ± 2.12 ^bc^	461.10 ± 19.09 ^b^	401.73 ± 20.51 ^c^	59.37 ± 1.41 ^d^	25.74 ± 1.32 ^bc^	25.74 ± 0.83 ^bc^	23.38 ± 0.81 ^b^	18.00 ± 1.41 ^ab^
	3	65.60 ± 1.41 ^c^	26.60 ± 0.71 ^bc^	39.00 ± 0.71 ^d^	392.05 ± 4.95 ^c^	337.25 ± 6.36 ^d^	54.80 ± 1.41 ^d^	22.65 ± 1.32 ^bc^	26.09 ± 0.83 ^b^	23.28 ± 0.81 ^b^	14.50 ± 0.71 ^cd^
T3	1	42.70 ± 3.54 ^d^	22.90 ± 2.12 ^cd^	19.80 ± 5.66 ^e^	255.80 ± 13.44 ^d^	231.10 ± 12.73 ^e^	24.70 ± 0.71 ^e^	22.65 ± 1.32 ^cd^	24.99 ± 0.83 ^bc^	19.98 ± 0.81 ^c^	11.00 ± 1.41 ^ef^
	2	40.40 ± 1.41 ^de^	21.30 ± 2.83 ^d^	19.10 ± 4.24 ^e^	233.60 ± 12.73 ^de^	210.43 ± 10.61 ^ef^	23.17 ± 2.12 ^e^	25.74 ± 1.32 ^d^	23.89 ± 0.83 ^cd^	22.68 ± 0.81 ^b^	12.50 ± 0.71 ^de^
	3	36.20 ± 0.71 ^e^	20.20 ± 3.54 ^d^	16.00 ± 2.83 ^ef^	206.97 ± 7.07 ^e^	184.23 ± 5.66 ^f^	22.74 ± 1.41 ^e^	16.89 ± 1.32 ^d^	23.29 ± 0.83 ^d^	18.28 ± 0.81 ^c^	13.50 ± 0.71 ^bc^
T4	1	22.70 ± 0.71 ^f^	14.90 ± 0.71 ^e^	7.80 ± 1.41 ^g^	91.30 ± 7.07 ^f^	76.60 ± 6.36 ^g^	14.70 ± 0.71 ^f^	12.65 ± 1.32 ^e^	14.99 ± 0.83 ^e^	9.98 ± 0.81 ^d^	9.00 ± 1.41 ^f^
	2	23.90 ± 4.95 ^f^	12.80 ± 0.71 ^e^	11.10 ± 5.66 ^fg^	79.60 ± 7.07 ^f^	67.42 ± 6.34 ^g^	12.19 ± 0.73 ^f^	15.74 ± 1.32 ^e^	13.89 ± 0.83 ^e^	12.68 ± 0.81 ^d^	8.00 ± 1.41 ^f^
	3	24.20 ± 2.12 ^f^	11.70 ± 1.41 ^e^	12.50 ± 3.54 ^fg^	77.47 ± 2.12 ^f^	64.40 ± 1.17 ^g^	13.07 ± 0.95 ^f^	16.89 ± 1.32 ^e^	13.29 ± 0.83 ^e^	14.28 ± 0.81 ^e^	8.50 ± 0.71 ^f^

Each value is Mean ± SD of 2 individual observations. Means in the same column sharing a same superscript letter are not significantly different and determined by DMRT test (<0.05) (TL—total length, GL: green length, RL—root length, TW—total weight, GW—green weight, RW—root weight, LW: leaf weight, LL—leaf length, LWI—leaf width, LN: no leaves).

**Table 7:** Biochemical composition of the iAVs-cultivated lettuce greens.

Experiment	Moisture (%)	Ash (%)	Protein (mg/kg)	Fiber (mg/kg)	Fat (mg/kg)	NFE (%)	Chlorophyll (mg/kg)
T1	96.23 ± 0.16 ^a^	20.53 ± 0.90 ^a^	28.70 ± 1.78 ^a^	12.13 ± 0.15 ^a^	2.83 ± 0.45 ^a^	33.33 ± 2.40 ^a^	1.40 ± 0.24 ^a^
T2	96.39 ± 0.05 ^a^	20.77 ± 1.14 ^a^	24.23 ± 0.91 ^b^	12.53 ± 0.80 ^a^	2.93 ± 0.81 ^a^	31.40 ± 2.31 ^a^	0.95 ± 0.02 ^ab^
T3	95.68 ± 0.24 ^a^	18.37 ± 1.63 ^b^	20.90 ± 0.70 ^c^	10.00 ± 0.78 ^b^	2.53 ± 0.35 ^a^	32.57 ± 1.48 ^a^	0.94 ± 0.45 ^ab^
T4	90.65 ± 1.56 ^b^	9.63 ± 0.59 ^c^	14.63 ± 0.81 ^d^	8.27 ± 1.05 ^c^	1.37 ± 0.12 ^b^	22.70 ± 1.39 ^b^	0.82 ± 0.14 ^b^

Each value is Mean ± SD of 2 individual observations. Means in the same column sharing the same superscript letter are not significantly different and determined by DMRT test (*p* < 0.05).

**Table 8:** iAVs cultivated lettuce leaves contained macro- and micro-mineral composition (ppm).

	Ca	Na	K	Mg	P	S	Co	Fe	Mn	Zn
T1	1.57 ± 0.06 ^a^	0.87 ± 0.08 ^d^	0.56 ± 0.05 ^a^	0.66 ± 0.04 ^a^	0.81 ± 0.05 ^a^	0.36 ± 0.02 ^a^	1.49 ± 0.60 ^a^	144.03 ± 25.25 ^a^	158.50 ± 37.69 ^a^	116.63 ± 5.56 ^a^
T2	1.52 ± 0.05 ^b^	1.58 ± 0.20 ^c^	0.42 ± 0.03 ^b^	0.59 ± 0.04 ^b^	0.78 ± 0.00 ^a^	0.35 ± 0.01 ^a^	1.10 ± 0.36 ^ab^	148.40 ± 31.21 ^a^	136.87 ± 18.39 ^a^	72.10 ± 9.03 ^b^
T3	1.34 ± 0.10 ^c^	2.59 ± 0.22 ^b^	0.36 ± 0.02 ^c^	0.41 ± 0.02 ^c^	0.68 ± 0.01 ^b^	0.29 ± 0.01 ^b^	0.69 ± 0.11 ^c^	83.43 ± 6.33 ^b^	91.50 ± 5.29 ^b^	42.93 ± 2.46 ^c^
T4	0.90 ± 0.13 ^d^	4.10 ± 0.08 ^a^	0.25 ± 0.03 ^d^	0.33 ± 0.02 ^d^	0.41 ± 0.04 ^c^	0.26 ± 0.01 ^c^	0.63 ± 0.02 ^c^	75.53 ± 22.64 ^b^	78.77 ± 3.48 ^b^	35.20 ± 1.56 ^c^

Each value is Mean ± SD of 2 individual observations. Means in the same column sharing the same superscript letter are not significantly different and determined by DMRT test (*p* < 0.05).

## Data Availability

Data are contained within the article.
